# Using EEG to decode semantics during an artificial language learning task

**DOI:** 10.1002/brb3.2234

**Published:** 2021-06-15

**Authors:** Chris Foster, Chad C. Williams, Olave E. Krigolson, Alona Fyshe

**Affiliations:** ^1^ Department of Computer Science University of Victoria Victoria Canada; ^2^ Centre for Biomedical Research University of Victoria Victoria Canada; ^3^ Departments of Computing Science and Psychology University of Alberta Edmonton Canada; ^4^ Alberta Machine Intelligence Institute (Amii) Edmonton AB Canada

**Keywords:** electroencephalography, language, language learning, machine learning, semantics

## Abstract

**Background:**

As we learn a new nonnative language (L2), we begin to build a new map of concepts onto orthographic representations. Eventually, L2 can conjure as rich a semantic representation as our native language (L1). However, the neural processes for mapping a new orthographic representation to a familiar meaning are not well understood or characterized.

**Methods:**

Using electroencephalography and an artificial language that maps symbols to English words, we show that it is possible to use machine learning models to detect a newly formed semantic mapping as it is acquired.

**Results:**

Through a trial‐by‐trial analysis, we show that we can detect when a new semantic mapping is formed. Our results show that, like word meaning representations evoked by a L1, the localization of the newly formed neural representations is highly distributed, but the representation may emerge more slowly after the onset of the symbol. Furthermore, our mapping of word meanings to symbols removes the confound of the semantics to the visual characteristics of the stimulus, a confound that has been difficult to disentangle previously.

**Conclusion:**

We have shown that the L1 semantic representation conjured by a newly acquired L2 word can be detected using decoding techniques, and we give the first characterization of the emergence of that mapping. Our work opens up new possibilities for the study of semantic representations during L2 learning.

## Graphical abstract

Using electroencephalography (EEG) and an artificial language that maps symbols to English words, we show that it is possible to use machine learning models to detect a newly formed semantic mapping as it is acquired.

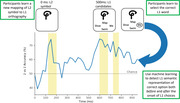

## 1 INTRODUCTION

Learning a new nonnative language (L2) is difficult and requires dedication and practice. Some of the first lessons when learning an L2 aim to teach a small basic vocabulary so that subsequent lessons can use that vocabulary. What happens in the brain during these first lessons, as we learn to map a familiar concept to a foreign word‐form? The neural representations evoked by a word‐form from a participant's native language (L1) have been studied extensively (Mitchell et al., 2008; Sudre et al., 2012; Wehbe et al., 2014; Huth et al., 2016), but little attention has been paid to the evoked semantic representations during the process of learning a new language.

Here we ask: can methodologies effective for detecting lexical semantics in an adult's L1 be used to detect newly mapped semantic meanings evoked by the L2? If so, what are the neural signatures of the newly mapped representation (e.g., timing of onset, salience, distribution over the scalp), and how might they differ from results for L1?

**FIGURE 1:**
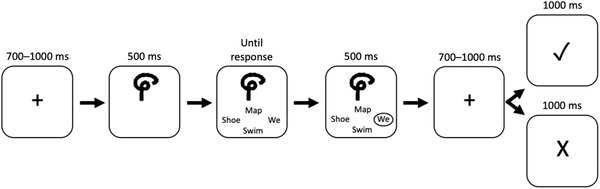
An overview of the experimental paradigm. Participants were required to learn a mapping of symbols to English words through trial and error to simulate vocabulary learning. Participants were shown a symbol, and then presented with four word‐forms from which to choose (here *map, shoe, we, swim*). Based on their response (here *we*), they receive positive or negative feedback about their selection. More details appear in Section 2.1

To answer these questions, we used a machine learning methodology developed to detect semantic representations in L1 (Sudre et al., 2012). Unlike ERP (event‐related potential) analyses, which compare the magnitude of electroencephalography (EEG) signals across conditions (e.g., N400, P600), our machine learning models allow us to detect multivariate patterns indicative of word meaning across multiple EEG sensors at multiple time points. This process of recovering aspects of the stimuli from brain images is called *decoding*. We trained and tested our machine learning models on signals collected via EEG during a language learning paradigm (see Figure 1). During the EEG experiment, participants learned a mapping of symbols to meanings through trial and error. Our results showed that

1. the acquisition of a new semantic mapping can be detected with EEG;
2. In order to obtain a reliable signature of semantic mapping, participants need to see the symbol several times, more than would be predicted based on behavioral measurements;
3. like seen previously for L1 representations, the neural representation of a newly learned symbol is detectable in many of the EEG sensors, but the representation may be shifted in time and less sustained than previously reported in L1; and
4. the semantics of a word can be decoded even when it is evoked by a totally arbitrary symbol.

Together, our results describe the characteristics of newly learned semantic mappings in the human brain and provide a paradigm and analysis framework for studying semantic representations during language learning.

### 1.1 Reading in a native language (L1)

During L1 reading, semantic incongruities in sentences (e.g., He spread the warm bread with *socks*.) can be detected based on ERP responses in EEG (Kutas & Hillyard, 1980; Kuperberg, 2007). Machine learning methods can decode word meaning from brain images using multivariate patterns, even when there is not a clearly visible separation in the univariate response (e.g., ERPs, activation of individual voxels). For example, machine learning can be used with functional magnetic resonance imaging (fMRI) data to differentiate between trials where participants view pictures or read sentences (Mitchell et al., 2002). Complex neuronal activation features can also be extracted using machine learning for tasks like detecting ambiguous sentences, performing sentiment analysis, or determining the category of an object (Mitchell et al., 2002; Shinkareva et al., 2008; Gu et al., 2014).

Additionally, machine learning models allow us to more deeply explore semantic processing and have allowed us to track the flow of information in the human brain during reading via fMRI and MEG (Mitchell et al., 2008; Sudre et al., 2012). Mitchell et al. showed that machine learning could be used to detect which word‐forms were being read by a participant using fMRI. By training a model to accurately predict the expected fMRI activity for a noun from the probability of collocation with 25 verbs, Mitchell et al. showed that the semantic features of a word (e.g., edibility, manipulability) are correlated with fMRI data of a participant reading that word (Mitchell et al., 2008). Specifically, a linear regression model was trained to use the probability of a word‐form being collocated with each of the 25 verbs to predict the fMRI activity. This prediction was then used as part of the 2 vs. 2 test (described in Section 2.3) to more robustly measure the efficacy of the models. Although their model was trained using 60 concrete nouns, the model was capable of generating predictions for thousands of words for which it had never seen corresponding fMRI data. This work differed from previous work, which performed statistical comparisons of brain activity across a small number of conditions (e.g., syntactically sound vs. malformed sentences) (Kutas & Hillyard, 1980; Kuperberg, 2007) or was only being capable of recognizing classes of brain activity that the model had encountered during training (Mitchell et al., 2002; Mitchell et al., 2002; Shinkareva et al., 2008; Gu et al., 2014). Mitchell et al. (2008) demonstrated a direct relationship between the statistics of word‐form co‐occurrence and the neural activation associated with each word's meaning.

Another key study in this area reproduced Mitchell et al. (2008) using MEG (Sudre et al., 2012). However, rather than generating features based on word‐form colocation, the Sudre et al. features were based on human ratings [1–5] for a set of semantic properties (e.g., Is it alive? Is it bigger than a golf ball?). Sudre et al. used a ridge regression model to predict the semantic property ratings from the MEG data. MEG allowed Sudre et al. to more accurately identify when in time the semantics of a word could be detected, and when the representation was the strongest. Subsequent work showed that, with some fine tuning, word vectors derived from a text corpus could be as accurate for predicting the word‐form a person is reading as the behavioral vectors used in Sudre et al. (Murphy et al., 2012). Word vectors are lists of numbers which abstractly represent the semantic relationships between words through the similarity and spatial relationships of word vectors (Mikolov et al., 2013b). For example, the vector for “king” minus the vector for “man” plus the vector for “woman” is similar to the vector for “queen” (Mikolov et al., 2013c). Such word vector models can be generated in many ways, but often are the by‐product of a model trained to estimate the probability of colocated words in a corpus, as is the case for Skip‐Gram (Mikolov et al., 2013a) (the word vector model used in this work).

Studying the brain's representation of individual word meanings has proceeded largely with MEG and fMRI; EEG has remained comparatively underutilized. This may be due to the challenges that come with EEG data (e.g., lower spatial resolution, comparatively poor signal‐to‐noise ratio). One of the first studies to successfully differentiate word meanings using and EEG was performed by  Murphy et al. (2009). In addition, they were able to distinguish between two semantic classes (land mammals or work tools) (Murphy et al., 2009, 2011). The model's accuracy was as high as 98% when averaged over multiple analyses, providing evidence that EEG could give more cost‐effective exploration of brain‐based semantics in more naturalistic environments. Our study adds to the body of evidence that EEG can be used to detect semantic representations with significant predictive accuracy.

One benefit that comes with MEG/EEG is better time resolution, allowing for a pinpointing in time of the onset of a semantic representation. Using MEG, Sudre et al. (2012) determined the early onset of decoding accuracy around 100 ms after the onset of the word‐form. Other studies corroborate this using combined EEG/MEG to showing that models can differentiate between semantic categories as early as 150 ms into the trial (Moseley et al., 2013). In our experiment, participants are shown the L2 symbol, followed by a set of four English words from which to choose the correct meaning. Thus, after the semantic mapping has been formed, we would expect to see a signature of the correct meaning after the onset of the symbol and possibly after the onset of the English choices (if the correct answer dominates the neural signature).

All reading paradigms suffer from visual confounds that arise because semantics and word‐form have correlations. For example, because some dimensions of a word vector model are correlated with the frequency of the corresponding word‐form (Hollis & Westbury, 2016), and shorter word‐forms are, on average, more frequent than longer ones (Piantadosi et al., 2011). Because longer word‐forms will also take up more space in the visual field and be made up of more white pixels, it is possible that some part of the visual signal alone could be used to predict the word vector without using semantic information. However, our paradigm uses a randomly selected symbol to represent word meaning, and so we are able to disentangle some of the visual effects present in typical word‐reading paradigms. Our word‐to‐symbol mapping is totally arbitrary, our paradigm triggers the semantics of the word without suffering from the typical *visual* confounds of the written word‐form.

### 1.2 L2 language learning

Our experimental design allows us to study participant learning in a unique way by applying a machine learning model that leverages word vector representations. However, learning more broadly has been traditionally studied in EEG using ERPs. One ERP of interest is known as the *reward positivity*, which is characterized as a frontal‐central (peaks at FCz and Cz) deflection 260–360 ms following feedback stimulus onset (Proudfit, 2015; Williams et al., 2021). The amplitude of the reward positivity is associated with behavior‐measured learning when presented in a reinforcement learning paradigm (Holroyd & Coles, 2002; Williams et al., 2017) (described in greater detail in Section 2.1). However, the exact nature of the reward positivity's association with learning remains unclear. In some work, the reward positivity is found to have a progressively reduced amplitude as participants perform better on the task and in other work, this correlation has not been consistently detected (Walsh & Anderson, 2012). Previous work exploring the same data analyzed here showed that neural signatures were indicative of learning and that they were indicative of behavioral measures of learning (Williams et al., 2020). Here we propose an alternative methodology for studying learning using a decoding approach.

Of course, language learning is very complex and requires more than vocabulary learning. Our study explores one of the first tasks in learning an L2, the acquisition of a basic meaning‐to‐word‐form mapping. A small learned vocabulary can then bootstrap incidental language learning, which may be a more effective mode for language learning (Huckin & Coady, 1999). Though gray matter and white matter changes have been documented over many weeks of language learning (Hosoda et al., 2013), the neural representations during the early stages of vocabulary learning have not been explored. It has been shown that EEG can detect when a participant is reading an unknown word‐form (Schneegass et al., 2019), but it is unclear if the signals of learning might dwarf those related to the semantic representation of a newly learned word. We do know that, once a person acquires an L2, the neural representations evoked by their L1 and L2 are very similar (Buchweitz et al., 2012).

This paper combines paradigms from the field of learning with methodology from previous studies of the brain's representation of meaning. We apply existing semantic decoding methodology (Mitchell et al., 2008; Sudre et al., 2012) to EEG data. We use a reinforcement learning task to guide participants as they learn an artificial language. To detect semantics, we trained a machine learning model to predict word vectors (derived from an artificial neural network, which is a type of machine learning model loosely inspired by biological neural networks) from raw EEG signal. This technique allows us to predict word meanings that were not included in our training set (as in  Mitchell et al., 2002).

## 2 MATERIALS AND METHODS

Our data were originally published by Williams et al. (2020). At this time, this research data are not publicly shared. The dataset consisted of EEG data from 25 undergraduate students (nine males) with an average age of 20 years (standard deviation 1.9 years), whom were recruited using an online sign‐up system to receive credit in a psychology course. Participants were not required to be native English speakers, but were required to be fluent in English. As such, our sample had an average self‐reported English fluency of 9.7 out of 10. In addition, the participants were unaware of the objectives of the current experiment. The task used here and by Williams et al. (2020) had participants learn a novel language by associating symbols to English word meanings. Williams et al. (2020) used this data to investigate trial‐by‐trial neural changes as reflected by the reward positivity and contrasted these trends to a reinforcement learning computational model. In other words, they investigated the neural underpinnings of learning curves. Here, we rather investigated the development of semantic maps across learning by incorporating the full range of EEG data within ridge regression models.

### 2.1 Paradigm

Participants viewed a series of symbols from the Tamil and Manipuri alphabets, each assigned to a random English word and associated meaning (consistent across all participants). Participants were presented with a symbol (considered the onset of a trial, 0 ms), and 500 ms later presented with four options from which to select the correct word‐form via a button press. The participant then received visual feedback about their response: (“✓” or “X”). The language learning here is not meant to be representative of learning a complete language, but is simply a proxy for early vocabulary learning.

Note that the stimuli used here were mapped to common English words. Participants were not learning new words in this paradigm, they were learning a mapping from unfamiliar symbols to familiar words in English.

To facilitate learning, symbols were selected from a set that grew as the experiment progressed. During the first block, participants were presented with six symbols (representing three pronouns, three verbs). In subsequent blocks, three new symbols (and thus three new word meanings) were added. These three new symbols were randomly paired with three previously seen symbols so that each block cycled through six symbols, and the randomness avoided confounds across participants. There were a total of 19 blocks, and 60 total symbols learned (see the Appendix for a full list). We chose this composition of word types because the original experiment also presented participants with sentences (e.g., I went store). The current article concerns the processing of words in general and thus all words were included in analyses, but no sentences. Because of the random addition of words throughout the experiment, each participant views each symbol for a different number of trials (ranging from 0 to 20).

The stimuli were displayed on a gray background. Each trial begins with a black fixation cross for 700–1000 ms, followed by a symbol written in black, 4.5 cm^2^ in size. The symbol presented was randomly selected from the list of six for the block. After 500 ms, four black English words appeared simultaneously in the arrangement of a fixation cross (top, bottom, right, left) below the symbol. One of the choices was the correct answer, and three distractor words (incorrect answers) were randomly chosen from the other five words in the block. The assignment of words to the four locations was randomly determined. Participants were instructed to respond as accurately as possible by pressing one of the buttons on the RESPONSEPixx controller, which also has response buttons arranged in a cross. Once a participant made a selection, the selected word turned white for 500 ms, the screen changed to a fixation for 700–1000 ms, and a feedback stimulus appeared for 1 s (“✓” or “X”). If a selection was not made within 2 s, an exclamation mark would appear to signify that they took too long to respond. (See Figure 1 for an overview of the paradigm and timing.) Within a block, participant accuracy was computed over a window of 10 symbols, and participants stayed on the current block until the participant received 90% or higher accuracy over a window of 10.

To further facilitate, the transfer of meaning to symbols, participants also viewed three‐word sentences beginning with a pronoun (I, we, you) followed by a verb, ending with either an adjective or noun (e.g., *I go fishing*). The sentence phases displayed three sentences before and after each word‐learning phase described above. In these phases, participants saw one symbol at a time for 1 s each, separated by a fixation cross for 700–1000 ms, which was followed by four English sentences from which to select what the sentence had said. For the purposes of our study, the sentence trials were discarded. The participants each saw on average 667 (σ=79) symbol exposures, including sentences, with breaks provided.

### 2.2 Data preprocessing

All EEG data were processed using Brain Vision Analyzer software (version 2.1.1, Brain Products GmbH, Munich, Germany). Data from each participant were manually reviewed to identify bad or flat channels due to a poor connection or movement. The channels were marked and removed from the dataset. The data were then downsampled from 500 to 250 Hz, re‐referenced to the average mastoid reference, and put through a dual pass phase free Butterworth filter (pass band: 0.1–30 Hz; notch filter: 60 Hz). Epochs were then extracted from the EEG data −1000 to 2000 ms around the onset of the symbol. The large time range was to facilitate the correction of eye blinks and movements artifacts via independent component analysis (ICA) provided by Brain Vision Analyzer (Luck, 2014). A restricted fast ICA with classic PCA (principal component analysis) sphering was used to identify components. This process continued until either a convergence bound of 1.0 × 10${}^{-7}$ or 150 steps had been reached. Ocular artifacts were selected manually by inspection of the component head maps and related factor loading and corrected via ICA back transformation. Electrodes that were initially removed were interpolated via spherical splines.

Symbol trials were then resegmented and trimmed to a 1000‐ms window following stimulus onset. The data were also baseline corrected using the 200‐ms prior to stimulus onset. Lastly, artifact rejection was applied. Any trial that contained an absolute difference between the lowest and highest voltage in that trial of more than 100μV for any electrode was discarded. Every trial that contained any period where the increase or decrease on any electrode was more than 10μV/ms was discarded as well. In total, 23% of the trials were discarded.

### 2.3 Methodology

Our methodology closely followed Sudre et al. (2012). We trained a series of ridge regression models that use the EEG data to predict the values for each dimension of the word vectors, a process often referred to as *decoding*. Our word vectors came from the Skip‐Gram model described by Mikolov et al. (2013a). Hollis et al. (2017) showed that Skip‐Gram vectors could be used to predict human judgments for semantic tasks (e.g., sentiment ratings), and Skip‐Gram vectors have been used to identify the semantics of many word types in fMRI , EEG, and MEG , and shown to perform similarly to other word vector models (Xu et al., 2016). Though the model's accuracy might change when using different word vectors, in our experience the general *patterns* of model accuracy (e.g., timing of peaks, spatial location) are less sensitive to the choice of word vector model.

Our EEG data have high dimension (many EEG sensor/time features) but comparatively few samples (words). In this scenario, regularization can improve model performance on held out data by shrinking the weights corresponding to irrelevant or less useful features, thereby reducing the number of effective parameters in the model. For example, in EEG data, signals originating from areas of the brain not engaged in the relevant language task are likely irrelevant. For this reason, we use ridge regression ($L_{2}$‐regularized regression), which robustly handles data of high dimension by encouraging near‐sparsity (very small learned weights for less useful features), and has a closed form solution (whereas $L_{1}$ regularized regression does not). In addition, in cases where features are noisy but correlated, $L_{2}$ regularization outperforms $L_{1}$ (Ng, 2004).^1^

**FIGURE 2:**
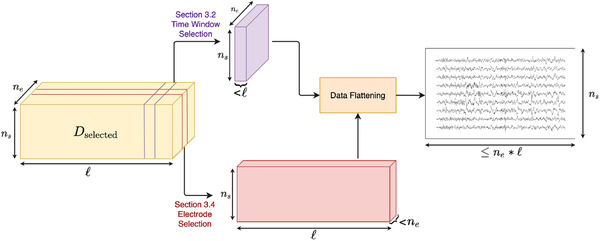
The data reshaping pipeline. Averaged trial data from $D_{\text{selected}}$ can be directly passed to the data flattening process, or it can be passed through a time window or electrode selection processes. These processes reduce either the number of time points (ℓ) or the number of electrodes ($n_{e}$) considered in downstream analysis. Though not pictured, we can also perform both time and electrode selections at the same time. We reshape the resulting tensor, flattening across the electrode dimension, such that the final data matrix has $n_{s}$ (number of symbols) rows and $\leq (n_{e}*l)$ columns

The EEG data for each symbol and each participant can be represented by a tensor^2^ $D\in R^{\left(r\times n_{e}\times l\right)}$, where r is the total number of times a symbol was seen by a participant. Because of the randomness of the paradigm, r ranges between $0\;\text{and}\;n_{t}$, where $n_{t}$ is the maximum number of possible trials seen for a given symbol. We use $n_{e}$ to denote the total number of electrodes, l for the number of time points, $n_{p}$ for the number of participants, and $n_{s}$ for the number of symbols. Depending on the type of analysis being performed, we selected some subset of trials, electrodes, or time points from D. We then averaged across all selected trials and participants to create a tensor of dimension $n_{s}\times n_{e}\times l$, denoted as $D_{\mathrm{selected}}$. Figure 2 gives an overview of the data selection and reshaping process. Using only a subset of electrodes/time points allows us to test for the presence of information at a particular location/time.

To accommodate the training of regression models, $D_{\text{selected}}$ was reshaped to produce a matrix with dimensions $X\in R^{n_{s}\times \left(n_{e}*l\right)}$. The Skip‐Gram word vectors of dimension v form a matrix $Y\in R^{n_{s}\times v}$. We trained v independent ridge regression models, such that each model predicts each dimension of the Skip‐Gram word vectors. We use a linear least squares loss function and L2‐norm regularization (ridge regression):

(1) $$\underset{W_{:,i}}{\min\;}||XW_{:,i}-Y_{:,i}{\left|\right|}_{2}^{2}+\alpha ||W_{:,i}{\left|\right|}_{2}^{2},$$

where regression model i is trained to predict the ith dimension of the word vectors (column vector $Y_{:,i}$) using weights $W_{:,i}$. The symbol : indexes every element in the dimension, indicating the selection of a whole row or column vector from a matrix. α is a hyperparameter that controls the level of regularization. Regularization helps to prevent overfitting and therefore increase the models' robustness to noise. We use a standard α=0.1, although we tested several values and found the only minor variation in performance. Using our trained regression model, we can predict a single element of a word vector for a given input $X_{i,:}$ via ${\hat{Y}}_{i,j}=X_{i,:}\cdot W_{:,j}$. Given the set of v regression models (parameterized by W), we can predict a full Skip‐Gram vector and thus approximate the meaning of the symbol from the EEG.

**FIGURE 3:**
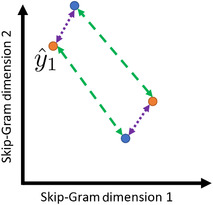
A visual explanation of the 2 vs. 2 test. The axes correspond to the first and second dimensions of the Skip‐Gram word vectors. The blue points ($y_{1}$ and $y_{2}$) correspond to the position of two hypothetical word vectors in this two‐dimensional space, the true word vectors. The orange points(${\hat{y}}_{1}$ and ${\hat{y}}_{2}$) represent the predictions from our ridge regression model for EEG collected while people viewed the symbols mapped to the two hypothetical word vectors. The 2 vs. 2 test measures the distance between matched (purple dotted line) versus mismatched (green dashed line) true‐to‐predicted assignments and chooses the assignment that minimizes the sum of the distances. Here, $y_{1}$ would be assigned to ${\hat{y}}_{1}$ and $y_{2}$ would be assigned to ${\hat{y}}_{2}$ because the purple lines are shorter than the green lines

We evaluated the set of ridge regression models in a “leave two out” fashion using the *2 vs. 2 test*. We held out pairs of symbols and trained ridge regression models to predict the vectors of the associated words using the EEG data from the remaining $n_{s}-2$ symbols. The model then predicts word vectors using the held out EEG data. In the 2 vs. 2 test, the true word vectors ($Y_{i,:}$, $Y_{j,:}$) are compared to the predicted word vectors (${\hat{Y}}_{i,:}$, ${\hat{Y}}_{j,:}$) using cosine distance. The 2 vs. 2 test passes if the sum of the distances between the correctly matched true and predicted word vectors ($d\left(Y_{i,:},{\hat{Y}}_{i,:}\right)+d\left(Y_{j,:},{\hat{Y}}_{j,:}\right)$) is smaller than the distance of the mismatched vectors ($d\left(Y_{i,:},{\hat{Y}}_{j,:}\right)+d\left(Y_{j,:},{\hat{Y}}_{i,:}\right)$). We ran the 2 vs. 2 test for all possible ns2 pairs of words. If there is no relationship between the EEG data and the word vectors, the 2 vs. 2 accuracy (the percentage of the ns2 2 vs. 2 tests correct) will be near chance (50%). For a visual depiction of the 2 vs. 2 test, see Figure 3.

We tested statistical significance using permutation tests. For each analysis, we reran the above pipeline, but randomly shuffled the order of the word vectors so that the true word vectors no longer correctly matched with the EEG data for each symbol. We corrected for multiple hypothesis testing using the Benjamini–Hochberg–Yekutieli procedure (Benjamini & Yekutieli, 2001) where applicable, using α=0.05.

## 3 EXPERIMENTS AND RESULTS

We devised four analyses to study the emergence of semantic representations in the brain during the symbol learning paradigm.

### 3.1 Semantic representation analysis

When the participants initially viewed the symbol, they did not know which English word meaning it mapped to. But, as they learned the symbol mapping through trial and error, they developed a semantic understanding. First, we tested the simple (but previously untested) hypothesis that EEG data collected while participants viewed a symbol can be used to predict the word vector for the corresponding English word.

For each participant, we removed symbols that were presented less than six times and removed the first two trials of each symbol (as symbols were rarely learned before the third trial). We tested using data from 0 to 500 ms after the onset of the symbol, which excludes the appearance of word choices at 500 ms. We then averaged the remaining trials over all participants for each symbol, which gave us a single noise‐reduced trial‐average per symbol. With this data, our regression models produced a 2 vs. 2 accuracy of 69.15%, which is statistically above chance (p<.001) and shows that the semantic representation for the L1 word is available after viewing the L2 symbol.

### 3.2 Time windowing analysis

We have determined that the semantic representation of the word can be detected using EEG collected during the time the symbol is visible on the screen. We also wished to understand the time course of semantic representation recall when evoked by a newly learned symbol. Here, we took advantage of EEG's high temporal resolution to analyze the brain's processing of symbols over time. We extended our windows of analysis out beyond the onset of the four word choices to see how the onset of word choices affects our ability to detect the semantics of the word. We evaluated the model pipeline on 50 ms windows of EEG data, which reduces the dimensions of $D_{\mathrm{selected}}$ to $R^{n_{s}\times \left(n_{e}*l_{s}\right)}$, $l_{s}\leq l$.

### 3.3 Onset of acquisition analysis

We have shown that semantic representation of learned symbols can be detected using EEG data collected after the second trial of a symbol. Now we ask, by what trial can we detect the semantic mapping? We averaged sets of three subsequent exposures in order to improve the signal to noise ratio and ran our decoding pipeline on that average. We compared the 2 vs. 2 accuracy for the earlier trials (e.g. trials 1–3) to the later trials (e.g. trials 4–6) to test if we can measure the emergence of a semantic mapping during the paradigm. As in Section 3.1, we only considered participant–symbol pairs with six or more exposures, to ensure an equal number of exposures are included in each group. We compared the 2 vs. 2 accuracy of averaged overlapping subsets of three exposures, selected from the first six exposures.

**FIGURE 4:**
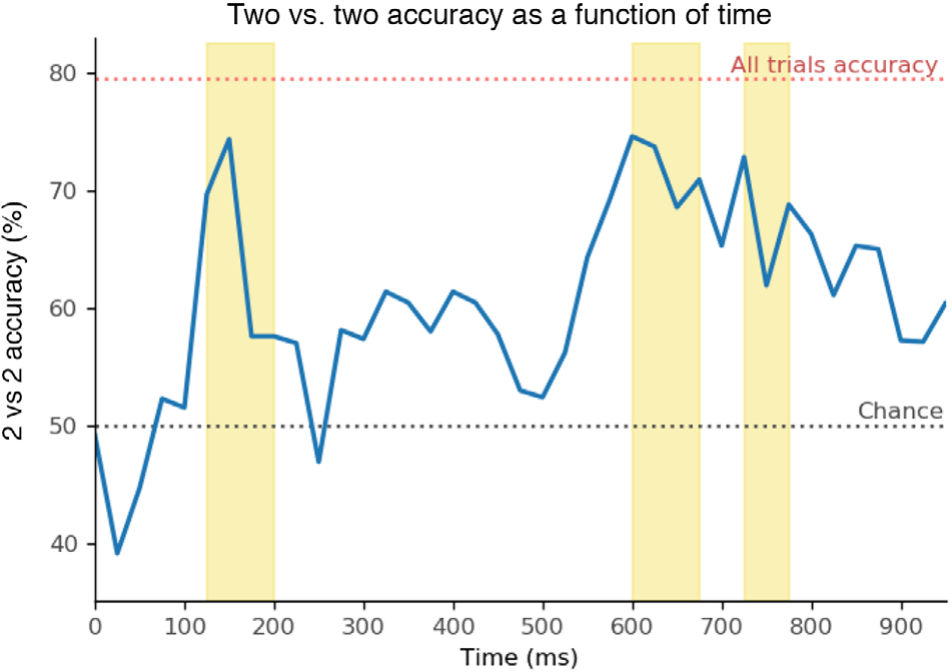
2 vs. 2 accuracy over time using 50 ms windows of EEG data. The *x*‐axis denotes window start point (e.g., 25 ms corresponds to a window 25–75 ms). Symbol onset is at 0 ms, word choices at 500 ms. Statistically significant windows are highlighted in yellow (p<.001, false discovery rate (FDR) corrected as described in Section 2.3)

**FIGURE 5:**
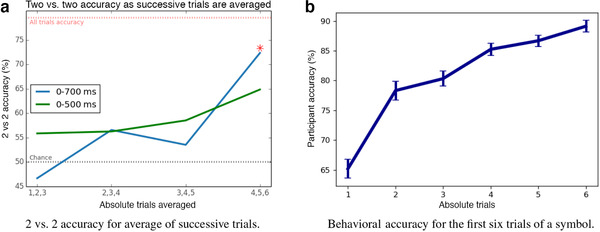
A comparison of behavioral and 2 vs. 2 accuracy. (a) The 2 vs. 2 accuracy when averaging three subsequent trials using two windows of time: 0–500 and 0–700 ms. 2 vs. 2 accuracy increases notably for the average of trials (4, 5, 6), showing that learning has occurred. The asterisk indicates the final value is significantly above chance (p<.001, FDR corrected). Error bars are not shown here, because data are averaged across participants before the 2 vs. 2 accuracy is computed. (b) Behavioral accuracy for the first six trials of a symbol. Error bars show standard error over the 25 participants. Assuming perfect learning, chance on the first trial should be 71%

Figure 5a shows the 2 vs. 2 accuracy over trials using a sliding window of three trails. Based on the results in Figure 4, we explored results for two time periods: 0–500 ms (containing only EEG signal collected before the onset of the word choices) and 0–700 ms (containing the above‐chance peak that occurred after the onset of the word choices). Though there is a general upward trend as the data are drawn from later trials, we only measured above chance 2 vs. 2 accuracy when using EEG data from trials (4, 5, 6) and the 0–700 ms time window. The 2 vs. 2 accuracy over trials (4–6) was slightly lower than the 2 vs. 2 accuracy reported in Section 3.1, because the latter analysis included trials beyond the sixth exposure. We applied bootstrapping to generate normal theory confidence intervals and confirmed a statistically significant difference in 2 vs. 2 accuracy between the first three trials (1–3) and the later trials (4–6) (p<.05) when using the 0–700 ms time window. Thus, our model can detect the onset of the acquisition of symbol meaning, but requires the more stable semantic representation that is present after the onset of word choices.

Figure 5b shows the participant response accuracy (using the button press) for the first six presentations of a symbol. If participants were perfect learners, we would expect to see participant accuracy of 71% for the first trial of a symbol, averaged over all symbols in all blocks. Because participants are not perfect, we observe a slightly lower 63% participant accuracy on the first trial. By the sixth trial, participants have an accuracy of 89%. Interestingly, the behavioral accuracy climbs much faster than our 2 vs. 2 accuracy using the EEG data. This implies that, while the participants may be reliably choosing the correct word choice by the second trial, the semantic representation is not consistent enough to be detected using EEG data until trials (4–6) are included. It is likely that many of the correct responses during the first few trials are correct guesses rather than recalled mappings, which is apparent when comparing to the 2 vs. 2 accuracy using EEG data. On correct guess trials, the semantic meaning would not have been available during the viewing of the symbol, as the symbol occurs before the onset of the word choices.

### 3.4 Electrode selection analysis

In addition to the timing of semantic representations, we were also interested in the *localization* of semantic representations. Thus, we explored the 2 vs. 2 accuracy using electrode groups, consisting of a central electrode and its immediate neighbors (to help combat noise). We analyzed three time windows: 0–500, 500–1000, and 0–1000 ms. We expected that the semantic representation would be very distributed, as previously reported (Murphy et al., 2011; Mitchell et al., 2008; Sudre et al., 2012; Huth et al., 2012, 2016).

**FIGURE 6:**
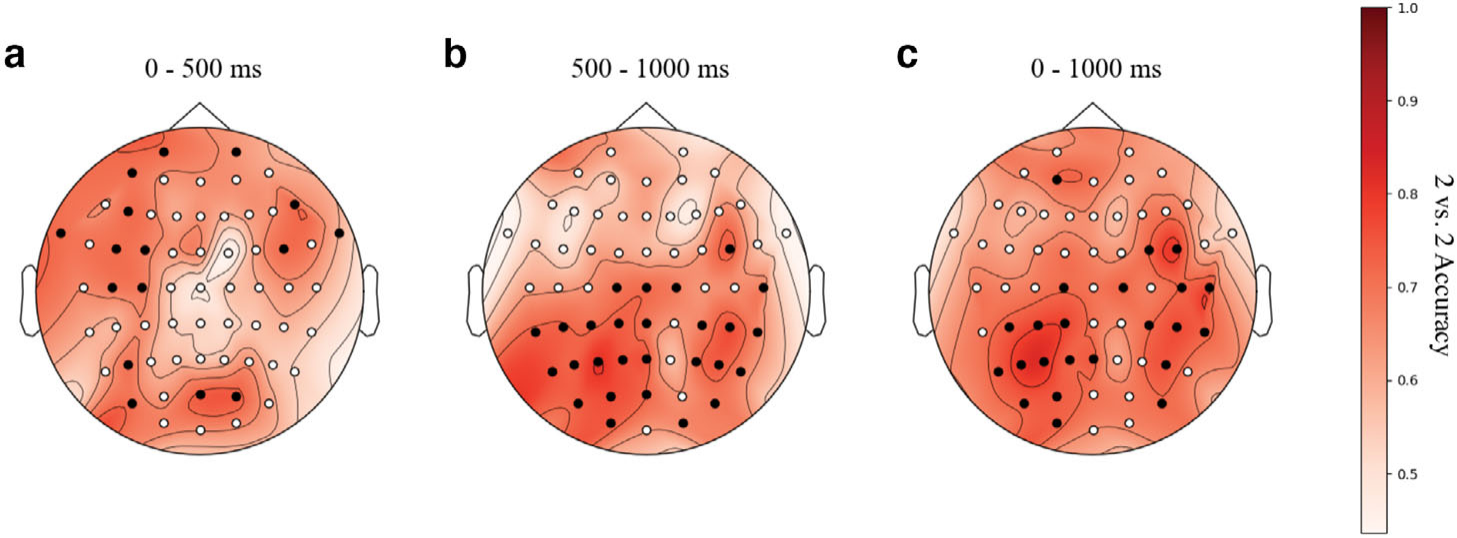
The results of a topographic analysis where each location represents an electrode and its immediate neighbors and color represent the magnitude of the 2 vs. 2 accuracy. (a) 0–500 ms window (only symbol visible); (b) 500–1000 ms window (only word choices visible); and (c) 0–1000 ms window. Statistically significant groups are shown as black circles (p<.001, FDR corrected)

The topographic interpolation of 2 vs. 2 accuracy for three time windows appears in Figure 6. We used the 2 vs. 2 accuracy of each electrode group to annotate the 2 vs. 2 accuracy of the central electrode, and electrodes above chance are shown as black circles. We saw lower 2 vs. 2 accuracies for the earlier time window (0–500 ms) compared to the later time window (500–1000 ms); however, we saw the highest accuracies over the entire time window (0–1000 ms). During the earlier time window, the left hemisphere dominates, which is reasonable given the left‐lateralization of language. Note that the topography of the 0–1000 ms window is very similar to the 500–1000 ms window, indicating that not much is gained by including the 0–500 ms time period.

## 4 DISCUSSION

We have shown that we can detect newly acquired semantic representations using EEG with statistically significant 2 vs. 2 accuracy. While previous work used the *reward positivity* ERP to show that learning can be detected via EEG (Williams et al., 2020; Krigolson et al., 2014), our work is the first to show that the *product* of that learning (i.e., the semantic representation) can be detected.

We were able to achieve a 2 vs. 2 accuracy of 69.15% in the semantic representation analysis, which provides evidence that there is a strong relation between the EEG data and the word vectors mapped to each symbol. This confirms our hypothesis that we would see statistically significant 2 vs. 2 accuracy. Previous decoding work has mostly focused on MEG and fMRI (Mitchell et al., 2008; Sudre et al., 2012), but our results show that a similar word decoding methodology can also be applied to EEG data even when the paradigm differs (i.e., during word learning). Murphy et al. (2009) explored decoding using EEG , but was focused on two semantic categories of concrete nouns (tools and mammals) while we expand to a much more varied vocabulary and utilize a different paradigm based on reinforcement learning. Though EEG has its limitations, it is sometimes preferable due to reduced cost and improved portability over MEG and fMRI. The adaption of this methodology to EEG an important contribution, as EEG lowers the costs for studying semantic representations by several orders of magnitude.

The 2 vs. 2 accuracy as a function of time *within* a trial showed two peaks, one during the 150–200ms window (74.34%) and one at 600–650 ms window (74.57%). For contrast, we explored the results described in the most similar previous work. However, it should be noted that none of these experiments match ours along all dimensions (words used, recording modality, word vectors, accuracy measure used, etc.). In particular, due to differences in signal quality inherent to the modalities (EEG signals are more spatially smooth), EEG‐based decoding accuracy is typically lower than with MEG. Thus, the following comparisons are meant only to give a sense of what the underlying patterns may be; it is not a properly controlled comparison.

In their MEG experiments, Sudre et al. (2012) found that the decodability of nouns rises above chance when using the window 50–100 ms after stimulus onset and peaks during the window 400–450 ms. Using EEG, and nouns from one of two semantic categories, Murphy et al. (2011) found that the Bhattacharyya metric of class separability in time‐frequency space peaked at 125 ms, and an optimal window for classification started at 100ms. We expected that the onset of above chance 2 vs. 2 accuracy would be later for our experiment, as participants must map each symbol to its English counterpart.

The onset of above‐chance 2 vs. 2 accuracy occurs during the 125–175 ms window. Thus, a delay may be present in our results. It is possible that using a newly learned semantic mapping slows the onset of a semantic representation by about 75 ms compared to MEG (Sudre et al., 2012), and 25 ms compared to EEG (Murphy et al., 2011). The peak of 2 vs. 2 accuracy actually occurs 250 ms *earlier* when compared to MEG, and 25 ms later in EEG. We hypothesize that we observed an earlier 2 vs. 2 accuracy peak not because the word meaning was recalled more quickly, but rather because the semantic representation is not as rich and complex as in the participant's L1, and so the neural activity is not sustained for the same length of time. Again, there are several differences between our experiments and this related work, so a more controlled experiment will be needed to verify these initial findings.

In our topographic analysis (Figure 6), during the 500–1000 and 0–1000 ms windows, we saw a more distributed pattern with a strong centroparietal component. Work using fMRI to study the representation of words from a participant's L1 has also found the highest scoring voxels were distributed across the cortex (Mitchell et al., 2008; Pereira et al., 2018). However, our results disagree with the spatial analysis by Sudre et al. (2012), who found that most ROIs (regions of interest) peak before 500 ms, with some exceptions in the temporal lobe or frontal lobes. Recall that our language‐learning paradigm is quite different than the single word viewing paradigm in Sudre et al. (2012), so these differences may stem purely from that. It may also be the case that the difference is partially attributable to the differences in spatial accuracy for MEG versus EEG. Or, it may be due to the additional work required to retrieve meaning via a newly learned symbol mapping, which could result in stronger activity in the left‐lateralized language areas of the brain. Alternatively, the differences may be due to the onset of word choices which appear at 500 ms. Reading the English word‐form at 500 ms may trigger a stronger, more distributed representation than reading the symbol. The response to the English word‐form may also be more strong because the mapping to meaning is more rote. It is also possible that, upon reading the word choices, the participant may confirm a mapping of which they were unsure, thus triggering a stronger response than the symbol. Further experiments are required to determine which of these factors contribute to the differences in the topography of decoding accuracy.

It is notable that our stimuli were symbols rather than word‐forms. Because of this, we were able to disentangle some of the visual effects present in typical reading paradigms. For example, word vectors are correlated with word frequency (Hollis & Westbury, 2016), and shorter words are, on average, more frequent than longer words (Piantadosi et al., 2011). Because longer words will also take up more space in the visual field and thus use more pixels, it is possible that the visual signal alone could be used to predict the word vector, thus inflating the 2 vs. 2 accuracy. Because frequency and length are correlated, it becomes difficult to remove this confound from a stimuli set. Our word‐to‐symbol mapping is totally arbitrary, and so our paradigm triggers the semantics of the word without suffering from the typical visual confounds of the written word‐form. However, there remains the possibility that participants are simply recalling the word‐form without recalling its semantic meaning, and that our models are thus able to decode based on a phonological representation that is available in the EEG signal, rather than semantic representations. Though we cannot rule this out, in previous work we have seen that, while word length does contribute to decoding accuracy, it does not explain all of the decoding accuracy (Fyshe et al., 2019).

Previous studies used written words alongside illustrations (Mitchell et al., 2008; Sudre et al., 2012), and recent work showed that decoding accuracy is higher when utilizing illustrations for word context (Pereira et al., 2018). Critiques of this earlier work have questioned if models were simply leveraging the brain's visual representations (e.g., dominant shapes or word length), suggesting that decoding accuracy might not be related to word meaning. Indeed, Sudre et al. (2012) determined the early onset of decoding accuracy they observed was correlated to the visual features of their stimuli (e.g. *word length* or *image diagonalness*). Still, other studies have also shown evidence of semantics earlier in time, such as (Moseley et al., 2013) who were able to differentiate between semantic categories as early as 150 ms into the trial .

Because our mapping of symbols to words was totally arbitrary, our results are strong evidence that word vectors truly correlate to the brain's representation of word *meaning*, and not some low‐level visual features of the stimuli. Although the mapped English word did become visible as one of the four options at the 500 ms mark, we saw statistically significant 2 vs. 2 accuracy even when training on a period when only the symbol is visible. It should be noted, however, that it is possible that in the recall of the word, there are phonological signals that could correlate to nonsemantic word features (e.g., word length). Thus, it is possible that our methods are simply decoding the phonological signature of the word. So, while we have removed the visual confound, it is not possible to completely eliminate all lower‐level word features from the detectable brain activity.

In previous work, participants were requested to visualize the concepts while viewing words and images (Mitchell et al., 2008; Sudre et al., 2012). In our experiment, participants were provided no instructions regarding visualization, only instructed to perform the reinforcement learning task. This provides evidence that the semantic representations are detectable in a more complicated symbol‐meaning mapping task, not only when performing a visualization task. This also shows that participants do not need to be coached to explicitly visualize the concepts in order to detect semantics.

Our work also shows that the word vector approach generalizes to different parts of speech. Many previous studies used only concrete nouns (Mitchell et al., 2008; Sudre et al., 2012; Murphy et al., 2009). Although the majority of our words were nouns, participants also saw adjectives, pronouns, and verbs. Even concrete and abstract nouns can have different electrophysiological attributes that make semantic modeling a more complicated task (Barber et al., 2013).

## 5 RELATED WORK

Machine learning models applied to fMRI and EEG have allowed us to track the flow of information in the human brain during word reading (Mitchell et al., 2008; Sudre et al., 2012). EEG data, however, have remained comparatively underutilized for the fine‐grained distinction of the meaning of individual words, possibly due to the challenges that come with EEG data (e.g., lower spatial resolution, comparatively poor signal‐to‐noise ratio). One of the first studies to successfully use word vectors to differentiate word meanings with EEG was performed by Murphy et al. (2009). In addition, they were able to distinguish between two semantic classes (land mammals or work tools) (Murphy et al., 2009, 2011). The decoding accuracy was as high as 98% when averaged over multiple analyses, providing evidence that EEG could give more cost‐effective exploration of brain‐based semantics in more naturalistic environments. Our study adds to the body of evidence that EEG can be used to decode fine‐grained semantic representations with significant 2 vs. 2 accuracy, even when those representations are evoked by a newly learned L2–L1 mapping.

Participant learning is often measured with the *reward positivity* ERP component (Proudfit, 2015; Krigolson et al., 2014). However, reward positivity does not always coincide with learning‐related behavioral changes (i.e., task accuracy), making it potentially unclear if reward positivity is related to a direct brain function related to learning, or if reward positivity is an indirect effect related to receiving feedback (Walsh & Anderson, 2012). Previous work exploring the same data analyzed here showed that ERP reward positivity signatures were at least indicative of behavioral measures of learning (Williams et al., 2020). Our work provides another angle for comparison, as we measured learning by detecting the actual *concept* to be learned (here, word meaning, as represented by a word vector) rather than measuring the neural response to reward via an ERP component. Since our model detects the semantic representation, it can be used to model both the process of learning and the later retention of learning, whereas the reward positivity only shows when learning has occurred. Thus, our approach could offer benefits in experiments where it is important to measure the *retention* of the mapping, or the robustness of the representation.

Language learning is very complex and requires more than vocabulary learning. Our study explores one of the first tasks in learning a new language, the acquisition of a basic set of words. Previous work has shown that once a person is fluent in an L2, the neural representation of a concept evoked by the two different languages are very similar (Buchweitz et al., 2012). Though gray matter and white matter changes have been documented over many weeks of language learning (Hosoda et al., 2013), the neural representations during the early stages of vocabulary learning may be difficult to interpret. Work with EEG has demonstrated the neural representation of language to match the complexity of language itself (Vandenberghe et al., 2019). The most widely studied ERP components of language are the N400 and the P600; however, language‐related effects have also been demonstrated in the N170, P300, and LPC (late positive complex) components (Vandenberghe et al., 2019). Together, these components reflect aspects of language including form, meaning, and use.

Indeed, language learning has been tracked with ERP components, showing that the ERP signatures of a new language converge towards that of the L1 after learning and consolidation (Vandenberghe et al., 2019). For example, there has been a documented shift in the electrophysiological correlates of language across learning; the N400 component is emphasized early in learning, and the P600 component is emphasized late in learning (Robert et al., 2018). As the N400 component is theorized to reflect semantic processing of language and the P600 component to reflect syntactic processing of language (Faretta‐Stutenberg & Morgan‐Short, 2018), these findings may imply that learning a novel language begins with semantic considerations and stabilizes with syntactic considerations.  (Morgan‐Shortt et al., 2010) had participants learn an artificial language and interpret sentence structures that varied on grammatical accuracy—half of the sentences were grammatically correct, while the other half presented syntactic violations (i.e., improper gender markings of adjectives or articles). They found an N400 to persist from early to late in learning, and the P600 to emerge throughout learning. Thus, these findings demonstrate the potential of EEG in the assessment of developing language capacities. Moreover, it was shown that EEG could detect when a participant is reading an unknown word (Schneegass et al., 2019), but it was unclear if the signals of learning might dwarf those related to the semantic representation of a newly learned word. To address the full complexity of neural signatures in language and language learning would then be to conduct a myriad of analyses (e.g., extract many different ERPs) across a range of tasks. Our work hints at a more succinct method for addressing the complex neural processes underpinning language and language learning.

## 6 CONCLUSION

EEG can be used to detect word meaning, even for a symbol‐based artificial language, and even during the process of language learning. We presented several decoding analyses using different time windows and electrode groups to help characterize the brain's representation of a newly learned symbol mapping. This hints at several new directions for studying brain function and the neural underpinnings of learning.

### PEER REVIEW

The peer review history for this article is available at https://publons.com/publon/10.1002/brb3.2234.

## Notes

1 Note that in this paragraph $L_{1}$ and $L_{2}$ correspond to the $L_{1}$ and $L_{2}$ norms applied to regression weights, not to native and nonnative languages (L1 and L2).

2 Whereas a matrix is a table of numbers with two dimensions (rows and columns), a tensor is an generalization of the matrix concept to include matrix‐like objects with an arbitrary number of dimensions. The tensors described here have three dimensions, like the yellow, purple, and red shapes in Figure 2.

## Word list

The full list of words used in this experiment is: angry, artist, be, book, cake, camping, car, cats, cinema, computer, dance, downtown, eager, education, energy, fishing, go, happy, have, hike, home, hungry, I, important, kind, late, library, map, market, mirror, money, musician, north, paper, pencil, phone, plan, poor, power, restaurant, rich, ring, run, sad, salesman, school, shirt, shoes, shopping, skate, smart, swim, teacher, thirsty, tired, travel, we, work, you, young.
